# Sex Differences in Two International Guidelines for Assessing Obstructive Coronary Artery Disease in Symptomatic Outpatients by Coronary Computed Tomographic Angiography

**DOI:** 10.31083/j.rcm2404101

**Published:** 2023-04-03

**Authors:** Yahang Tan, Zhe Wang, Qian Xin, Na Li, Fang Liu, Qiaoyu Xu, Mulei Chen

**Affiliations:** ^1^Heart Center and Beijing Key Laboratory of Hypertension, Beijing Chaoyang Hospital, Capital Medical University, 100020 Beijing, China; ^2^Senior Department of Cardiology, The Sixth Medical Center of PLA General Hospital, 100048 Beijing, China; ^3^Department of Radiology, Beijing Chaoyang Hospital, Capital Medical University, 100020 Beijing, China

**Keywords:** stable chest pain, sex differences, risk assessment, coronary computed tomography angiography

## Abstract

**Background:**

Low-risk individuals are unlikely to benefit from noninvasive testing, and women tend to have a lower prevalence of coronary artery disease (CAD). This study compared the performance of two current guidelines that differ by sex to assess s a'q's't chest pain outpatients, including symptom-based (2016 National Institute for Health and Care Excellence, NICE) and risk-based strategies (2019 European Society of Cardiology, ESC).

**Methods:**

A total of 542 outpatients referred for coronary computed tomography angiography (CCTA) at a single-centre were retrospectively included in this study. A risk assessment was calculated for each outpatient according to the two guidelines. Patients were classified into low and high-risk groups according to each strategy. The presence of coronary artery disease was the endpoint. Net reclassification improvement (NRI) was used to assess the performance of the two strategies.

**Results:**

The prevalence of CAD was 27%. The sensitivity, specificity, positive predictive value and negative predictive value for ESC and NICE were 90.4%, 54.3%, 42.2%, 93.9% and 78.8%, 35.6%, 31.1% and 82.0% respectively. Compare to NICE, the NRI for ESC were 30.32%. The ESC guidelines classified 55.56% of women and 28.14% of men into the low-risk group. The ESC guidelines had a higher predictive value for coronary artery disease compared to the NICE guidelines, with a positive NRI in men (15.55%) and women (34.46%) respectively.

**Conclusions:**

The ESC guidelines offered a more accurate calculation of risk assessment than the NICE guidelines. Patient sex influenced applying the recent ESC guidelines, which would result in a significant decrease in inappropriate testing of women but an increase in appropriate noninvasive testing of men.

## 1. Introduction

Stable chest pain suggestive of coronary artery disease (CAD) is a common symptom encountered by outpatients worldwide. To identify or exclude potential CAD, medical resources are the cornerstone of the diagnosis and clinical management of these patients [1]. Among these outpatients, women tend to have a lower likelihood of CAD than men. Therefore, some trials suggest the need for a sex-specific evaluation and diagnosis of CAD [2, 3, 4, 5]. Guidelines recommend estimating the pre-test probability (PTP) of CAD to optimize the balance between safety and efficiency of testing [6, 7].

Two distinct approaches have been independently released by the 2016 U.K. National Institute of Health and Care Excellence (NICE) and the 2019 European Society of Cardiology (ESC) [8, 9]. The 2016 NICE guidelines were updated with two important changes in which the PTP-based risk assessment was abandoned and noninvasive testing for myocardial ischaemia was replaced with broad indications for coronary computed tomography angiography (CCTA). The 2019 ESC guidelines introduced a new PTP score classifying patients into low and high-risk groups. Additional risk factors, such as the coronary calcium score (CCS), were considered to recalculate the clinical likelihood of CAD in patients with borderline PTP.

However, to the best of our knowledge, no study has compared the sex-related differences in the two approaches for stable chest pain patients. Thus, in this study, we validated and compared the relative accuracy for estimating CAD using the NICE and ESC strategies by CCTA in men and women, respectively.

## 2. Materials and Methods

**Fig. 1.**
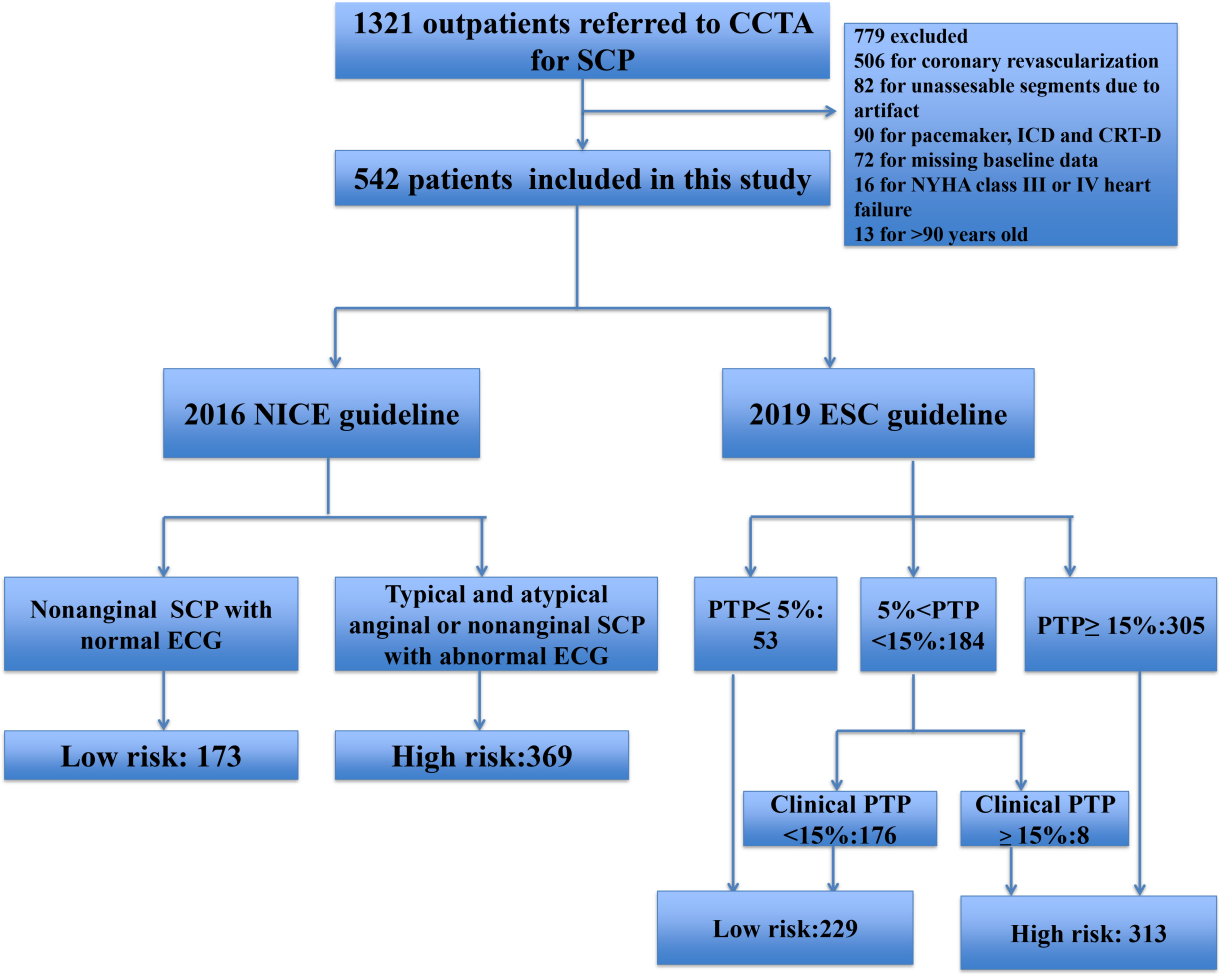
**Flow chart illustrating study population**. CCTA, Coronary Computed Tomography Angiography; SCP, Stable Chest Pain; ICD, Implantable Cardioverter defibrillator; CRT, Cardiac Resynchronization Therapy; NYHA, New York Heart Association; NICE, National Institute for Health and Care Excellence; ESC, European Society of Cardiology; PTP, Pre-test Probability; ECG, electrocardiogram.

We enrolled 1321 stable chest pain patients suspected of CAD who were referred for CCTA in a single regional cardiovascular centre recognized as tertiary A level (Beijing Chaoyang Hospital, Capital Medical University, Beijing, China) from August 2018 to December 2018. Among these patients, 779 patients were excluded (Fig. 1).

This study was carried out according to the code of ethics of the World Medical Association (Declaration of Helsinki); patients were provided written informed consent prior to inclusion in the study. The relevant protocols were approved by the Ethics Committee of Beijing Chaoyang Hospital.

### 2.1 Risk Assessment Approaches

Based on the clinical data and other information, a risk assessment of the enrolled participants was evaluated based on each strategy. Details of the risk groups of these two different risk assessment approaches are illustrated in Fig. 1 and as follows.

Participants were categorized into two groups for the 2016 NICE guideline analyses; the low-risk group included patients with non-anginal symptoms and a normal electrocardiograph (ECG), and the high-risk group included those with either typical or atypical chest pain or non-anginal symptoms with an abnormal ECG [9] .

Patients with PTP ≤5% were classified into the low-risk group and patients with PTP ≥15% were classified into the high-risk group for the 2019 ESC guideline analyses. The clinical likelihood of CAD was further calculated for patients with PTP between 5% and 15%, according to the recent ESC guidelines. We selected the CAD Consortium extended model, which incorporated clinical variables and CCS for further assessment. Based on previous research, only 2% of patients with PTP <15% had obstructive CAD based on the CAD consortium extended model [10]. Thus, in our study, patients with borderline ESC-PTP (5–15%) and clinical PTP <15% were categorised into the low-risk group, and the remainder were categorised into the high-risk group [8]. The differences between NICE and ESC in the Table 1.

**Table 1.** **The differences between NICE and ESC**.

	2016 NICE	2019 ESC
Factors	Nature of anginal symptoms, ECG	Age, sex, nature of anginal symptoms
Risk assessment	Low risk: Nonanginal SCP with normal ECG;	Low risk : PTP ≤5%;
	High risk: Typical and atypical anginal or nonanginal SCP with abnormal ECG	Moderate risk:
		5%< PTP <15%;
		High risk: PTP ≥15%
Referral for diagnostic testing	Low risk: No testing;	Low risk: No testing;
	High risk: CCTA	Moderate risk: Further clinical likelihood of CAD;
		High risk: Non-invasive evaluation
Further assessment factors	No	CCS, ECG, Risk factors for CAD (dyslipidaemia, diabetes, hypertension, smoking, family history of CAD), LV dysfunction suggestive of CAD
Further assessment	No	Clinical likelihood of CAD
		Low risk ≤5%, defer testing;
		High risk ≥15%, Non-invasive evaluation

NICE, National Institute of Health and Care Excellence; ESC, European Society of Cardiology; ECG, electrocardiograph; SCP, Stable chest pain; PTP, pre-test probability; CCS, coronary calcium score; CCTA, coronary computed tomography angiography; CAD, coronary artery disease.

### 2.2 CCS + CCTA

All patients underwent CCTA using a third-generation dual-source CT (DSCT) (SOMATOM Force; Siemens Healthineers, Forchheim, Germany). Sublingual nitroglycerine and heart-rate control for a target heart rate of at least 70 beats/min were administered as appropriate. The scanning parameters for DSCT were as follows: 2 × 64 × 0.6 mm acquisition collimation with the z-flying focal spot technique. Automated tube current modulation (Care Dose 4D, Siemens Healthcare) was used in all examinations. One tube of DSCT system was operated with 444 reference mAS per rotation at 70 kV, and the other tube was automatically operated with 127 reference mAs per rotation at 150 kV. All the scans were performed in cranio-caudal direction with patients in supine position during midispiratory breath-hold.

A non-contrast cardiac CT scan was acquired before CCTA. The CCS was calculated using Agatston software in Siemens Syngo Via VB20 (Siemens Healthineers, Erlangen, Germany). The presence of obstructive CAD was defined as the site interpretation of ≥50% according to the Coronary Artery Disease-Reporting and Data System [11].

### 2.3 Statistical Analyses

Data are presented as mean ± standard deviation for continuous variables and as frequencies for categorical variables. Continuous variables were compared using Student’s *t*-test or the Mann-Whitney U-test. Categorical variables are expressed as frequencies and percentages. Differences in categorical data were analysed with the chi-square or Fisher’s exact tests, as appropriate. The net reclassification improvement (NRI) estimate in the reclassification table was used to determine how the 2019 ESC guidelines reclassified patients into different risk groups compared to the 2016 NICE guidelines. All data analyses were performed using SPSS 22.0 software (IBM Corp., Armonk, NY, USA). A *p*-value < 0.05 (two-tailed) was considered significant.

## 3. Results

### 3.1 Study Population and Baseline Clinical Characteristics

As illustrated in Fig. 1, 542 outpatients were recruited for the final analyses, and 31.7% (172/542) were assigned to the low-risk group according to the 2016 guidelines. For the 2019 ESC guidelines, of the 184 patients with 2019 ESC pre-test probability between 5% and 15%, 176 had a clinical likelihood of CAD <15%. Together with the 53 patients with an ESC PTP <5%, the ESC strategy classified 42.3% (229/542) into the low-risk group.

Table 2 shows the sex-specific baseline characteristics of the outpatients. Men were more likely to smoke and have obstructive CAD than women (45% vs. 28%; 38% vs. 17%). The differences in age and the CCS were significant between men and women (63 ± 12 vs. 61 ± 11; 14.75 [0–212.25] vs. 0 [0–83.95]). The prevalence rates of diabetes, hypertension, hyperlipidaemia, family history of CAD, changes on ECG and type of angina were similar in men and women.

**Table 2.** **Baseline characteristics**.

Characteristics		Total (N = 542)	Men (N = 263)	Women (N = 279)	*p* value
Age (years)		62 ± 12	63 ± 12	61 ± 11	0.033*
Diabetes		144 (27)	61 (23)	83 (30)	0.098
Hypertension		360 (66)	174 (66)	186 (67)	0.928
Hyperlipidemia		179 (33)	87 (33)	92 (33)	1
Smoking		198 (37)	119 (45)	79 (28)	0*
Family history		88 (16)	43 (16)	45 (16)	1
Changes in ECG		147 (27)	69 (26)	78 (28)	0.699
Angina					0.406
Nonanginal		200 (37)	98 (37)	102 (37)	
	Atypical	246 (45)	109 (41)	137 (49)	
	Typical	96 (18)	56 (21)	40 (14)	
CCS		3.9 (0–152.6)	14.75 (0–212.25)	0 (0–83.95)	0*
	0	250 (46)	102 (39)	148 (53)	
	1–99	131 (24)	66 (25)	65 (23)	
	100–399	96 (18)	57 (22)	39 (14)	
	≥400	65 (12)	38 (14)	27 (10)	
Obstructive CAD detected by CCTA		146 (27)	99 (38)	47 (17)	0*

Values are presented as n (%) and mean ± SD. CCS, coronary calcium score; CAD, coronary artery disease. *was considered statistical significance.

The CCTA results revealed that greater than half of the outpatients had non-obstructive or no CAD, and 27% had obstructive CAD. Compared to patients in the low-risk group based on the ESC guidelines, patients in the high-risk group were more likely to have obstructive CAD (ESC guidelines: 42% vs. 6%, odds ratio [OR] 11.20, 95% confidence interval [CI]: 6,24–20.11, *p *< 0.001*). The difference between the high- and low-risk groups for obstructive CAD was significant when applying the NICE strategy after adjustment by gender, age, hypertension, hyperlipidemia,diabetes mellitus, smoke and family history of CAD (31% vs. 18%, OR 2.43, 95% CI: 1.47–4.03, *p* = 0.001*).

The CCTA results were similar for male outpatients as in the overall outpatients. About 63% of male outpatients had non-obstructive or no CAD and 38% had obstructive CAD. More than 80% of female outpatients had non-obstructive or no CAD detected by CCTA and 17% had obstructive CAD. The prevalence of obstructive CAD between the high- and low-risk groups of male and female was similar to the overall outpatients (male ESC guidelines: 48% vs. 11%, OR: 7.66, 95% CI: 3.49–16.83, *p *< 0.001*; male NICE guidelines after adjustment by age, hypertension, hyperlipidemia, diabetes mellitus, smoke and family history of CAD: 44% vs. 26%, OR: 2.09, 95% CI: 1.14–3.82, *p* = 0.017; female ESC guidelines: 33% vs. 4%, OR: 12.27, 95% CI: 5.00–30.11, *p *< 0.001*; female NICE guidelines after adjustment by gender, age, hypertension, hyperlipidemia, diabetes mellitus, smoke and family history of CAD: 20% vs. 9%, OR: 3.82, 95% CI: 1.43–10.20, *p* = 0.008*).

### 3.2 Reclassification Risk Assessment Strategies in Overall Outpatients

Table 3 showed the classification of all outpatients into risk categories (low risk and high risk) based on 2016 NICE and 2019 ESC guidelines. Of the 396 negative outpatients, 112 were reclassified correctly to low risk by the 2019 ESC guidelines, and 23 of the 146 positive outpatients were reclassified correctly as high risk. Thus, the NRI for the 2019 ESC guidelines was 18.68% for negative, 11.64% for positive, and 30.32% overall compared to the 2016 NICE guidelines (*p *< 0.05*).

**Table 3.** **Reclassification table comparing risk assessment strategies in overall outpatients**.

		Risk groups by 2019 ESC strategy		Total	Reclassification*		${\mathrm{NRI}}^{†}$	*p*
		Low	High		Up	Down		
Risk groups by NICE strategy								
Negative patients					9.60%	28.28%	30.32%	<0.05*
	Low	103	38	141				
	High	112	143	255				
	Total	215	181	396				
Positive ${\mathrm{patients}}^{‡}$					15.75%	4.11%		
	Low	8	23	31				
	High	6	109	115				
	Total	14	132	146				

NICE strategy, 2016 National Institute of Health and Care Excellence guideline-determined risk assessment strategy; ESC strategy, 2019 European Society of Cardiology guideline-determined risk assessment strategy; NRI, net reclassification improvement. *The reclassification of patients by the horizontal strategy was compared to that by the vertical one. ^†^NRI = [P(Up — Positive) – P(Down — Positive)] – [P(Up — Negative) – P(Down — Negative)]. ^‡^A positive patient was defined as a patient had obstructive CAD. *was considered statistical significance.

### 3.3 Reclassification Risk Assessment Strategies in Male Outpatients

The results were different for the risk analyses of men (Table 4). Of the 99 positive men, 19 were reclassified correctly as high risk by the 2019 ESC guidelines, whereas 3 were reclassified as low risk. Thus, the NRI for the 2019 ESC guidelines was –0.61% for negative, 16.16% for positive, and 15.55% overall compared to the 2016 NICE guidelines (*p *< 0.05).

**Table 4.** **Reclassification table comparing risk assessment strategies in male outpatients**.

		Risk groups by 2019 ESC strategy		Total	Reclassification*		${\mathrm{NRI}}^{†}$	*p*
		Low	High		Up	Down		
Risk groups by NICE strategy								
Negative patients					17.68%	17.07%	15.55%	<0.05*
	Low	38	29	67				
	High	28	69	97				
	Total	66	98	164				
Positive ${\mathrm{patients}}^{‡}$					19.19%	3.03%		
	Low	5	19	24				
	High	3	72	75				
	Total	8	91	99				

NICE strategy, 2016 National Institute of Health and Care Excellence guideline-determined risk assessment strategy; ESC strategy, 2019 European Society of Cardiology guideline-determined risk assessment strategy; NRI, net reclassification improvement. *The reclassification of patients by the horizontal strategy was compared to that by the vertical one. ^†^NRI = [P(Up — Positive) – P(Down — Positive)] – [P(Up — Negative) – P(Down — Negative)]. ^‡^A positive patient was defined as a patient had obstructive CAD. *was considered statistical significance.

### 3.4 Reclassification Risk Assessment Strategies in Female Outpatients

Table 5 showed the classification of women based on the two sets of guidelines. Of 232 negative women, 84 were reclassified correctly to low risk, whereas 4 were classified to high risk by the 2019 ESC guidelines. Thus, the NRI for the 2019 ESC guidelines was 32.33% for negative, 2.13% for positive and 34.46% overall compared to the 2016 NICE guidelines (*p *< 0.05).

**Table 5.** **Reclassification table comparing risk assessment strategies in female outpatients**.

		Risk groups by 2019 ESC strategy		Total	Reclassification*		${\mathrm{NRI}}^{†}$	*p*
		Low	High		Up	Down		
Risk groups by NICE strategy								
Negative patients					3.88%	36.21%	34.46%	<0.05*
	Low	65	9	74				
	High	84	74	158				
	Total	149	83	232				
Positive ${\mathrm{patients}}^{‡}$					8.51%	6.38%		
	Low	3	4	7				
	High	3	37	40				
	Total	6	41	47				

NICE strategy, 2016 National Institute of Health and Care Excellence guideline-determined risk assessment strategy; ESC strategy, 2019 European Society of Cardiology guideline-determined risk assessment strategy; NRI, net reclassification improvement. *The reclassification of patients by the horizontal strategy was compared to that by the vertical one. ^†^NRI = [P(Up — Positive) – P(Down — Positive)] – [P(Up — Negative) –P(Down — Negative)]. ^‡^A positive patient was defined as a patient had obstructive CAD. *was considered statistical significance.

## 4. Discussion

In this CCTA-based analysis of stable chest pain outpatients, women and men differed in the smoking and CCTA results: men were more likely to smoke and have obstructive CAD detected by CCTA. In addition, the low-risk group in the recent ESC guidelines indicated no CAD and the high-risk group was more likely to have CAD detected by CCTA compared to the NICE groups. The ESC strategy performed better than the NICE strategy with a positive NRI in outpatients. However, the reclassification of risk assessment between females and males was different. Using the ESC guidelines instead of the NICE guidelines would accurately decrease the risk classification and CCTA testing in females. It would accurately increase the risk classification and CCTA test for males. To the best of our knowledge, this is the first comparative description of a sex-based calculation of risk classification according to the 2016 NICE and 2019 ESC guidelines.

Consistent with previous investigations, we found that women were more likely to present with atypical angina and have a lower prevalence of obstructive CAD than men. In the promise study, men were more likely than women to characterize their chest pain as “aching/dull” and “burning/pins and needles”. Women were more likely than men to have back pain, neck, or jaw pain and palpitations as the primary presentating symptoms, men were more likely to have fatigue and/or weakness. The prevalence of typical and atypical between female and male patients was not different. However, women were more likely to present with nonanginal. In the recent ISCHEMIA trial, women had more frequent angina, but there was not detailed information on type of angina [4, 12, 13, 14]. Women did not have a larger burden of traditional risk factors, except for age and smoking, than men, suggesting that demographic risk factors may fail to influence the CAD prediction. As a novel imaging predictor of cardiovascular risk, the CCS was higher in male outpatients than female outpatients. Thus, these data suggest that different risk assessment models may have sex-specific performance for outpatients with stable chest pain, and incorporating the CCS may offer a more accurate risk classification.

The updated pre-test probabilities of CAD published by the 2019 ESC guidelines have been adjusted substantially downward and highlight the new concept of the clinical likelihood of CAD, particularly in patients with borderline PTP compared to the 2013 ESC guidelines. We noted that the new PTP recommended by the ESC improved the accuracy of the prediction for obstructive and non-obstructive CAD in all patients compared to the 2016 NICE strategy.

The 2019 ESC PTP improved the risk stratification through different mechanistic pathways in men and women compared to the 2016 NICE strategy in our study. The ESC PTP showed an NRI of 15.55% in men, which was ascribed to reclassification of 19.19% of men with positive CCTA to high risk, whereas 36.21% of women with negative CCTA were reclassified into low risk, resulting in an NRI of 34.46%.

The superiority of the ESC guidelines is ascribed to applying the clinical likelihood of CAD and incorporating traditional risk factors and the CCS, particularly the CCS. The CCS derived from routine cardiac-gated non-contrast CT has undergone extensive validation as a predictor of cardiovascular risk [15, 16, 17]. First, the distribution of the CCS differed by sex. Men had a higher CCS than women in our study, which was consistent with the results from the Multi-Ethnic Study of Atherosclerosis (MESA) [18]. The distribution of CCS features resulted in a risk reclassification in men and women. In our investigation, 19.19% of men with a positive CCTA result were reclassified into high risk and 36.21% of women with a negative CCTA result were reclassified into low risk. Second, a zero CCS in a patient with stable chest pain was associated not only with a very low prevalence of obstructive CAD but also with excellent long-term survival [19, 20]. In the present study, 53% of female outpatients had zero CCS and 36.21% of women with a negative CCTA result were reclassified into the low-risk group. However, a negative CCTA result was detected in only 39% of male outpatients, and 17.07% of men with a negative CCTA result were reclassified into the low-risk group.

The 2016 NICE guidelines recommend CCTA to assess and diagnose stable chest pain patients as the first test based on angina symptoms and discard the previous emphasis on calculating PTP. However, whether it should be universally accepted to evaluate stable chest pain in patients remains controversial. In our study, the performance of the NICE strategy was suboptimal compared to the recent ESC guidelines. Of the several explanations for the unsatisfactory risk assessment of the NICE strategy, the following two emerge as particularly strong candidates. First, the nature of symptoms alone is not a strong predictor of obstructive CAD. Although typical angina is associated with the highest prevalence of CAD, patients with atypical or no angina were likely to have >10% obstructive CAD.The symptom category showed no relationship to the prevalence of obstructive CAD in patients <40 years of age. Furthermore, the effect of the sex difference on predicting obstructive CAD should not be ignored. In the CONFIRM study, CAD severity was higher in men than women for every symptom category, which was similar to the PROMISE trial [12, 21, 22]. We also demonstrated that men were more likely to have a higher prevalence of obstructive CAD than women. This may attenuate the accuracy of the risk assessment according to the NICE strategy based on the nature of the anginal symptoms.

Several limitations must be considered in our analyses. This was a retrospective single-centre study and some data were not documented. There was selection bias resulting from different reasons for referral for CCTA, which limits generalizability. Information about dyspnoea calculated in the PTP and recommended in the 2019 ESC guidelines was missing, which may have caused us to overestimate the PTP. Thus, further multicentre and prospective studies are needed. In addition, obstructive CAD (≥50%) detected by CCTA was the gold standard test rather than invasive coronary angiography. Previous investigations have demonstrated that CCTA has a high negative predictive value but a lower positive predictive value [1, 23]. Finally, the effect of PTP on the follow-up downstream management and outcomes including prescriptions, referrals for noninvasive and invasive imaging testing, coronary revascularization and major adverse cardiovascular events were not included in this study because some data were lacking.

## 5. Conclusions

In conclusion, the 2019 ESC guidelines offered a more accurate calculation of the risk assessment than the 2016 NICE guidelines. The risk assessment model recently recommended by these two guidelines differed significantly by sex in outpatients presenting with stable chest pain and referred for CCTA. Applying the recent ESC guidelines instead of the NICE guidelines resulted in a significant downregulation of risk and a decrease in appropriate testing in women; however, it upregulated risk and increased appropriate noninvasive imaging in men.

## Data Availability

All data generated or analyzed during the current study are included in this article.
